# Estetrol Inhibits the Prostate Cancer Tumor Stimulators FSH and IGF-1

**DOI:** 10.3390/jcm13195996

**Published:** 2024-10-08

**Authors:** Herjan J. T. Coelingh Bennink, Erik P. M. Roos, R. Jeroen A. van Moorselaar, Harm H. E. van Melick, Diederik M. Somford, Ton A. Roeleveld, Tjard D. de Haan, Yacov Reisman, Iman J. Schultz, Jan Krijgh, Frans M. J. Debruyne

**Affiliations:** 1 Pantarhei Oncology, 3700 AL Zeist, The Netherlands; ijs@pantarheioncology.nl (I.J.S.); jk@pantarheibio.com (J.K.); 2 Department of Urology, Antonius Hospital, 8601 ZK Sneek, The Netherlands; e.roos@antonius-sneek.nl; 3 Department of Urology, Amsterdam UMC, Free University, 1081 HV Amsterdam, The Netherlands; rja.vanmoorselaar@amsterdamumc.nl; 4 Department of Urology, St. Antonius Hospital, 3435 CM Nieuwegein, The Netherlands; h.van.melick@antoniusziekenhuis.nl; 5 Department of Urology, Canisius-Wilhelmina Hospital, 6532 SZ Nijmegen, The Netherlands; r.somford@cwz.nl; 6 Department of Urology, North-West Hospital, 1815 JD Alkmaar, The Netherlands; t.a.roeleveld@nwz.nl; 7 Department of Urology, Isala Hospital, 8025 AB Zwolle, The Netherlands; t.d.de.haan@isala.nl; 8 Flare-Health, 1012 DA Amsterdam, The Netherlands; reismancobi@gmail.com; 9 Andros Clinics, 6803 AA Arnhem, The Netherlands; f.debruyne@andros.nl

**Keywords:** androgen deprivation therapy (ADT), PCombi study, estetrol (E4), follicle-stimulating hormone (FSH), insulin-like growth factor-1 (IGF-1)

## Abstract

**Background:** The co-treatment of androgen deprivation therapy (ADT) for advanced prostate cancer (PCa) with the fetal estrogen estetrol (E4) may further inhibit endocrine PCa tumor stimulators. We previously reported the suppression of follicle-stimulating hormone (FSH), total and free testosterone, and prostate-specific antigen by ADT+E4. Here, we provide more detailed data on FSH suppression by E4 and present new findings on the effect of ADT+E4 on insulin-like growth factor-1 (IGF-1). **Methods:** A Phase II, double-blind, randomized, placebo-controlled study (the PCombi study) was conducted in advanced PCa patients treated with ADT. The study assessed the effect of E4 co-treatment with LHRH agonist ADT on tumor stimulators, including FSH and IGF-1. Patients starting ADT were randomized 2:1 to receive either 40 mg E4 (*n* = 41) or placebo (*n* = 21) for 24 weeks. Non-parametric analyses were performed on the per-protocol population (PP) and individual changes were visualized. **Results:** The PP included 57 patients (37 ADT+E4; 20 ADT+placebo). ADT+E4 almost completely suppressed FSH in all patients (98% versus 37%; *p* < 0.0001). IGF-1 levels decreased by 41% with ADT+E4 versus an increase of 10% with ADT+placebo (*p* < 0.0001). **Conclusions:** The almost complete suppression of the tumor stimulator FSH using ADT plus E4 observed in all individual patients in this study, along with the augmented suppression of IGF-1 versus an increase by ADT only, may be clinically relevant and suggest the enhanced anti-cancer treatment efficacy of E4 in addition to the previously reported additional suppression of total and free T and PSA.

## 1. Introduction

Treatments that suppress and inactivate the androgen testosterone (T) are the cornerstone of prostate cancer (PCa) treatment. Until the 1970s, advanced PCa was treated with estrogens such as diethylstilbestrol, but these were abandoned because of major cardiovascular events (MACE) [1]. Thereafter, androgen deprivation therapy (ADT) with gonadotrophin-releasing hormone (GnRH) analogs (parenteral agonists and oral and parenteral antagonists) became available, as well as anti-androgenic drugs such as the androgen receptor signaling inhibitors (ARSI’s) [2]. The GnRH analogs are continued lifelong, except when used with radiotherapy for locally advanced PCa. Before the GnRH analogs were available, oral estrogens had been an effective treatment option for 30 years, reducing T-levels to inhibit the growth of androgen-sensitive PCa [3,4]. Although the efficacy of estrogens and GnRH analogs as ADT was comparable, the estrogens used in the past had major MACE and the replacement of estrogens with GnRH analogs was for safety and not for efficacy reasons [4]. However, this treatment paradigm change from estrogens to GnRH analogs created the problem of estrogen deficiency. By suppressing T, its metabolite estradiol (E2) is also decreased by about 80%, causing serious estrogen deficiency side-effects affecting quality-of-life (QoL) including arthralgia, hot flushes, weight gain, muscle atrophy (sarcopenia), bone loss and fractures, metabolic syndrome, increased arterial MACE risk, fatigue, sleeping problems, loss of energy, apathy, mood changes and depression, and cognition and memory problems (Table S1). Estrogen treatment with diethylstilbestrol (DES), ethinylestradiol (EE) or oral E2 has been associated with an increased risk of MACE [4,5]. However, new safer estrogen therapies including transdermal E2 (tE2) [6] and oral fetal estrogen estetrol (E4) [7,8,9] have shown promising anti-tumor effects, either in combination with ADT (E4) or without ADT (tE2). The reconsideration of the use of these safer estrogens is warranted, especially because these compounds prevent/treat estrogen deficiency signs and symptoms.

Estetrol is a natural human estrogen, produced by the fetal liver during pregnancy only [10]. Oral administration of E4 has a low impact on coagulation and hemostatic liver factors at doses up to 20 mg for hormone replacement therapy (HRT) [11]. The phase II PCombi study (co-treatment of PCa with ADT and oral E4) was conducted to assess the efficacy and safety of 40 mg E4 in patients with advanced PCa who started ADT treatment with an LHRH agonist (LHRHa) [7,8]. We previously reported that, compared to placebo, E4 co-treatment (ADT+E4) was well tolerated, and was associated with improvements in estrogen-deficiency symptoms. Moreover, ADT+E4 more potently suppressed the endocrine parameters total and free T, prostate-specific antigen (PSA), and follicle-stimulating hormone (FSH), consistent with enhanced anti-tumor effects [7,8]. Here, we present new findings on the effect of ADT+E4 on insulin-like growth factor-1 (IGF-1) and provide more detailed data on its FSH suppression. The important role of these two tumor stimulators is generally underestimated [12,13]. In a prospective analysis of 17,000 PCa cases, IGF-1 was positively associated with the risk of overall, aggressive, and early-onset disease [14]. There is a consensus that FSH plays a role in both the development and progression of PCa and its cardiometabolic comorbidities [13,15]. Moreover, it is considered that FSH has potential mutagenic effects in PCa [16].

## 2. Patients and Methods

### 2.1. Patients

Male patients with a body mass index of 18.0–35.0 kg/m^2^ who were recently diagnosed with locally advanced or metastatic PCa and who qualified for treatment with an LHRHa as ADT were eligible. In addition, patients had to have an Eastern Cooperative Oncology Group (ECOG) performance status of 0–1 [17] and a life expectancy of at least 2 years. The supplementary information provides a full list of the inclusion and exclusion criteria.

### 2.2. Study Design and Treatments

A randomized, double-blind, placebo-controlled, phase II, proof-of-concept study in patients with advanced PCa (PCombi) was conducted at four sites in the Netherlands (ClinicalTrials.gov NCT03361969; EudraCT 2017-003708-34) [7]. Patients were randomized at baseline at a 2:1 ratio to receive once-daily oral co-treatment of ADT with 40 mg E4 or matching placebo for a treatment duration of 24 weeks (weeks).

The study was approved by an independent ethics committee (Evaluation of Ethics in Biomedical Research, Assen, The Netherlands) and was conducted in accordance with the Declaration of Helsinki and the Good Clinical Practice guidelines of the International Council for Harmonization. All patients provided written informed consent.

### 2.3. Endpoints and Assessments

The endpoints were FSH and IGF-1 plasma concentrations as assessed using electrochemiluminescence immunoassay that was performed at the BARC Central Laboratory (Ghent, Belgium). Blood samples were taken at baseline and at 12 and 24 weeks.

### 2.4. Statistical Analysis

Analyses were performed on the per-protocol (PP) population. Baseline demographic data were compared using a chi-square exact test or *t*-test. Mean changes at 24 weeks with ADT+E4 compared to those with ADT+placebo were evaluated using the Kruskal–Wallis non-parametric test. To underscore the clinical relevance of the results beyond statistical significance, individual changes from baseline for each patient are also presented.

## 3. Results

In total, 63 patients were randomized and 62 received study medication (41 ADT+E4 and 21 ADT+placebo). The PP consisted of 57 patients: 37 on E4 and 20 on placebo (Figure S1 CONSORT diagram). Five patients discontinued treatment early, three due to adverse events (Figure S1). The majority of patient characteristics were similar between the treatment groups with the exception of BMI and ECOG performance status (Table 1).

**Figure 1:**
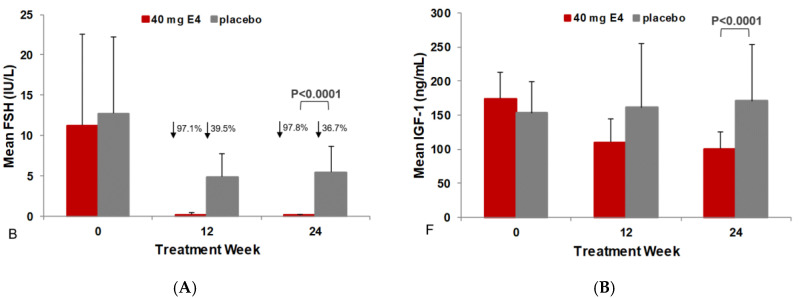
The mean levels of follicle-stimulating hormone (**A**) and insulin-like growth factor-1 (**B**) after 12 and 24 weeks of treatment with 40 mg estetrol or placebo in patients with prostate cancer treated with an GnRH agonist (per-protocol population). FSH: follicle-stimulating hormone; IGF-1: insulin-like growth factor-1; GnRH: gonadotrophin-releasing hormone; NB: No statistical analysis was performed on the 12-week data.

**Figure 2:**
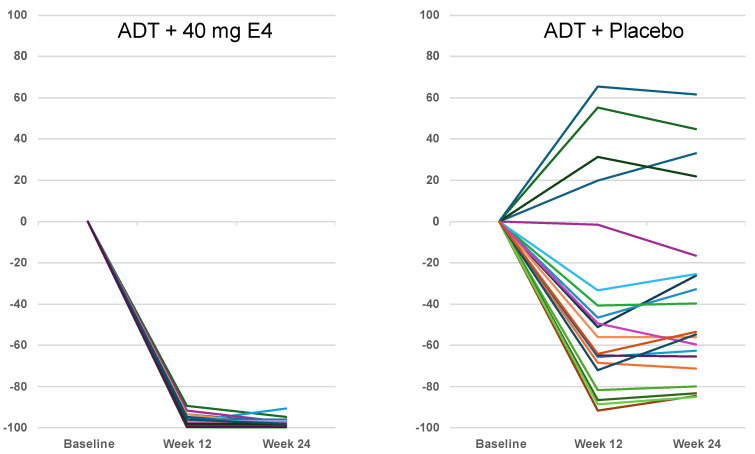
Individual change (%) from baseline follicle-stimulating hormone levels at weeks 12 and 24 of treatment with 40 mg estetrol or placebo ADT co-administration (per-protocol population). ADT androgen deprivation therapy; E4: estetrol; source: Supplementary Table S2.

ADT+E4 decreased the mean level of FSH from 11.3 ± 11.3 IU/L at baseline to 0.2 ± 0.3 IU/L at 12 weeks and remained at that low level at 24 weeks (−97.8%). With ADT+placebo, FSH levels decreased from 12.7 ± 9.5 IU/L to 4.9 ± 2.9 IU/L at 12 weeks and to 5.5 ± 3.2 IU/L at 24 weeks (−36.7%) (*p* < 0.0001) (Figure 1A, Table 2 and Table 3). The individual data show that FSH was almost completely suppressed in all patients treated with ADT+E4. With ADT+placebo, FSH decreased much less than with ADT+E4 and only in 16 of 20 patients (Figure 2, Table S2).

**Figure 3:**
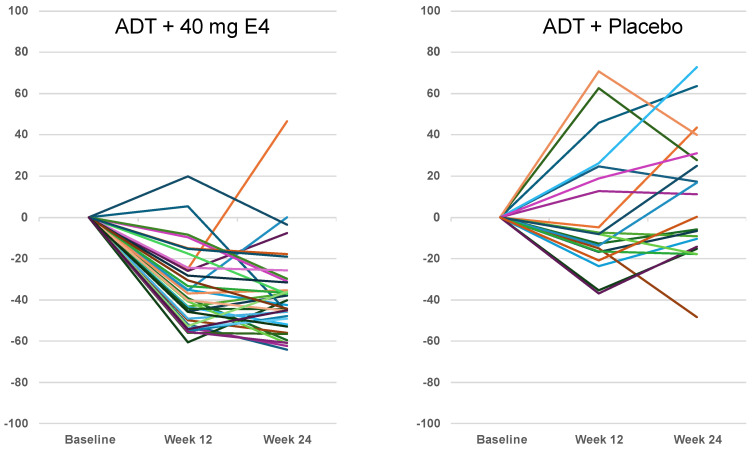
Individual change (%) from baseline insulin-like growth factor-1 levels at weeks 12 and 24 of treatment with 40 mg estetrol or placebo ADT co-administration (per-protocol population). ADT androgen deprivation therapy; E4: estetrol; source: Supplementary Table S3.

ADT+E4 decreased the mean level of IGF-1 from 174.0 ± 38.2 ng/mL at baseline to 110.2 ± 34.2 ng/mL (−35.8%) after 12 weeks treatment and to 100.4 ± 25.8 ng/mL (−41.4%) after 24 weeks. In contrast, the mean levels of IGF-1 increased slightly in the placebo group, from 153.1 ± 46.7 ng/mL at baseline to 161.3 ± 94.5 ng/mL (+2.1%) and 171.2 ± 82.3 ng/mL (+10.2%) at 12 and 24 weeks, respectively. The differences between treatment groups were significant (*p* < 0.0001) (Figure 1B, Table 2 and Table 3). The individual data show that at week 24, ADT+E4 suppressed IGF-1 levels in all but one of the patients, whereas with ADT+placebo, IGF-1 levels showed an about equal number of cases with an increase (*n* = 11) or a decrease (*n* = 9) of IGF-1 at 24 weeks (Figure 3, Table S3).

The PCombi study is rather small, but does not give rise to safety concerns. MACEs occurred more frequently in the placebo group (two cases, i.e., 9.5% of the placebo population) than in the E4 group (two cases, i.e., 4.9% of the E4 population) [7].

## 4. Discussion

Publications reporting clinical trials generally do not include individual effects. The size of the study reported in this paper, with 37 PCa patients treated with ADT+E4 and 20 patients treated with ADT+placebo, allows to show individual changes in the endocrine tumor stimulators investigated. While increased IGF-1 levels were observed in 10 out of 20 patients with ADT+placebo (+10.2% on average), all but one of the patients with ADT+E4 showed a decrease (−41.4% on average) (Figure 3, Table 3). An almost complete suppression of FSH was observed with E4 in all patients (−97.8% on average), whereas a suppression to a lesser extent was observed in 14 out of 20 patients (−36.7% on average) in the placebo group (Figure 2, Table 3). The mean data for FSH reaching significance (*p* = 0.0001) at 24 weeks in favor of ADT+E4 were reported earlier [7]. As known, changes versus baseline at 24 weeks for free T (*p* = 0.04) and PSA (*p* = 0.003) were also significant in favor of E4, while for total T suppression this occurred earlier (*p* = 0.05) [7]. These differences are reflected in the individual changes in these parameters (Figures S2–S4, Tables S4–S6). Altogether, these observations confirm that E4 demonstrated favorable effects in all E4-treated patients on the endocrine tumor stimulators that were investigated.

The principle aim of ADT is to reduce the circulating levels of T to castrate levels of total T to less than 0.6 nmol/L (20 ng/dL) [18,19], which was achieved in all patients in both treatment groups from week 4 through week 24 in this study [7]. The significant inhibition of free T, which is considered a better predictor than total T for castration resistance [20], can be explained by the increase in sex hormone-binding globulin by estrogens such as E4. The rather strong and significant effect of E4 on PSA is a new observation. PSA is kallikrein-3, a protease enzyme, that stimulates the growth of PCa [21], and the rise in PSA means a higher production of this tumor stimulator, which explains that the suppression of the tumor stimulator by ADT is associated with clinical benefits [22] and also explains why PSA is a marker for PCa tumor progression.

The mitogenic effect of FSH plays an important stimulating role in the development and progression of PCa [13,15,16]. In the male, FSH is the prime inducer of proliferation of spermatogonia and sperm production [23]. FSH levels are suppressed by ADT alone [13] and by ADT combined with an ARSI [24], or by tE2 only [25]. The almost complete suppression of FSH (97.8%) in all patients co-treated with E4 is therefore a significant anti-tumor effect, similar to the significantly enhanced suppression of the mitogen IGF-1. High levels of serum IGF-1 and activated IGF-1 receptor (IGF-1R) in the prostate are found in PCa patients [12]. Not only enhanced serum IGF-1, but also the activation of IGF-1R and its downstream signaling components have been increasingly recognized to play a vital role in driving the development of PCa [12]. Treatment modalities targeting IGF-1 are potential strategies for cancer therapy [12], and the observed effects of E4 in the PCombi study suggest a favorable effect on IGF-1.

New endocrine treatments for advanced PCa are generally based on a novel mode of action to inhibit or antagonize testosterone, all at the expense of the concomitant loss of estrogens. This results in a loss of QoL due to serious subjective side effects including hot flushes and arthralgia and objective symptoms such as bone loss and fractures, arterial vascular disease, and unfavorable brain effects. In the past, estrogens have been used very effectively for PCa treatment but have been replaced by LHRHas because of an increased risk of MACE. So far, the risk of MACE when using tE2 or E4 seems to be lower than the risk associated with older estrogens used in the past [5,6,7,9,26].

## 5. Limitations

The current and previously reported findings are robust for the enhanced suppression of IGF-1, FSH, total T, free T, and PSA during E4 co-treatment with ADT. In addition, we believe that the study population is similar to that of other well-designed ADT studies in PCa patients [27]. However, larger and longer-duration studies are needed to confirm that these secondary tumor suppression endpoints are predictive and representative for a better progression-free survival (PFS) and overall survival (OS) in men with advanced prostate cancer.

## 6. Conclusions

The almost complete suppression of the tumor stimulator FSH by ADT plus E4 that was observed in all individual patients in this study, along with the augmented suppression of IGF-1 versus an increase by ADT only, may be clinically relevant and suggest the enhanced anti-cancer treatment efficacy of E4 in addition to the previously reported additional suppression of total and free T and PSA.

## Figures and Tables

**Table 1:** Patient demographics and baseline characteristics (per-protocol population).

Parameter	40 mg Estetrol (*n* = 37)	Placebo (*n* = 20)	*p* Value *
Median age (range), yrs	74 (59–85)	75 (49–84)	NS
Weight, kg	82.9 (12.2)	90.0 (14.8)	NS
BMI, kg/m^2^	26.1 (3.4)	28.2 (3.7)	0.045
Tumor stimulator, Mean (SD) Total T (nmol/L) Free T (pmol/L) PSA (ng/mL) IGF-1 (ng/mL) FSH (IU/L)	19.4 (8.5) 42.0 (17.4) 18.4 (22.1) 174.0 (38.2) 11.3 (11.3)	19.1 (6.6) 37.7 (15.6) 33.5 (57.0) 153.1 (46.7) 12.7 (9.5)	NS NS NS NS NS
Duration since PC diagnosis, *n* (%) 0–3 months 3–6 months 6 months–1 yr >1 year	21 (56.8) 3 (8.1) 2 (5.4) 11 (29.7)	12 (60.0) 3 (15.0) 1 (5.0) 4 (20.0)	NS
Distant metastasis, *n* (%) M0 M1 M1a M1b M1c	25 (67.6) 7 (18.9) 2 (5.4) 3 (8.1)-	14 (70.0) 2 (10.0) - 3 (15.0) 1 (5.0)	NS
Gleason score, *n* (%) 6 7 ≥8	2 (5.4) 15 (40.5) 20 (54.1)	1 (5.0) 8 (40.0) 11 (55.0)	NS
ECOG performance status, *n* (%) 0 1	33 (89.2) 4 (10.8)	12 (60.0) 8 (40.0)	0.010
Previous prostatectomy, *n* (%)	9 (24.3)	3 (15.0)	NS
Radiotherapy for primary tumor, *n* (%)	7 (18.9)	2 (10.0)	NS

Results are expressed in mean (standard deviation) or otherwise specified. * For categorical variables, chi-square exact test was used; for continuous variables, the *t*-test was used [*p* value presented if statistically significant, otherwise as NS (not statistically significant)]. BMI: body mass index; ECO: Eastern Cooperative Oncology Group; FSH: follicle-stimulating hormone; IGF-1: insulin-like growth factor-1; PC: prostate cancer; PSA: prostate-specific antigen; T: testosterone; yr(s): year(s).

**Table 2:** Mean levels of IGF-1 and FSH at baseline and after 12 and 24 weeks of treatment (per-protocol population).

Parameter (Unit)	ADT+40 mg Estetrol (*n* = 37)			ADT+Placebo (*n* = 20)		
	Baseline	Wk 12	Wk 24	Baseline	Wk 12	Wk 24
IGF-1 (ng/mL)	174.0 (38.2)	110.2 (34.2)	100.4 (25.8)	153.1 (46.7)	161.3 (94.5)	171.2 (82.3)
FSH (IU/L)	11.3 (11.3)	0.2 (0.3)	0.2 (0.1)	12.7 (9.5)	4.9 (2.9)	5.5 (3.2)

Values are presented with standard deviation in brackets. ADT: androgen deprivation therapy; IGF-1: insulin-like growth factor-1; FSH: follicle-stimulating hormone.

**Table 3:** Suppression of IGF-1 and FSH after 24 weeks of treatment (per-protocol population).

Parameter (Unit)	ADT+40 mg Estetrol (*n* = 37)	ADT+Placebo (*n* = 20)	*p* Value *
IGF-1 (ng/mL)	−41.4 (15.3)	+10.2 (30.4)	<0.0001
FSH (IU/L)	−97.8 (1.7)	−36.7 (44.8)	<0.0001

Values are presented as the percentage change in the mean with standard deviation in brackets. ADT: androgen deprivation therapy; IGF-1: insulin-like growth factor-1; FSH: follicle-stimulating hormone. * Kruskal–Wallis test comparing week 24 laboratory levels of 40 mg estetrol with placebo.

## Data Availability

Individual data regarding the tumor stimulators investigated are presented in the Supplementary Materials. Other data are available from the corresponding author upon request.

## Supplementary Materials

The following supporting information can be downloaded at https://www.mdpi.com/article/10.3390/jcm13195996/s1: Figure S1—Disposition of patients (CONSORT); Figure S2—Individual change (%) from baseline of total testosterone levels at Weeks 2, 6, 12 and 24 of treatment with 40 mg estetrol or placebo ADT co-administration (per-protocol population); Figure S3—Individual change (%) from baseline of free testosterone levels at Weeks 2, 6, 12 and 24 of treatment with 40 mg estetrol or placebo ADT co-administration (per-protocol population); Figure S4—Individual change (%) from baseline of prostate-specific antigen levels at Weeks 2, 6, 12 and 24 of treatment with 40 mg estetrol or placebo ADT co-administration (per-protocol population); Table S1—Side effects of androgen deprivation therapy with, in brackets, whether it is due to the loss of testosterone (T) or estrogens (E); Table S2—Individual follicle-stimulating hormone (FSH) levels throughout the study (per-protocol population), and change (%) from baseline; Table S3—Individual insulin-like growth factor-1 (IGF-1) levels throughout the study (per-protocol population), and change (%) from baseline; Table S4—Individual total testosterone (T) levels throughout the study (per-protocol population), and change (%) from baseline; Table S5—Individual free testosterone (T) levels throughout the study (per-protocol population), and change (%) from baseline; Table S6—Individual prostate-specific antigen (PSA) levels throughout the study (per-protocol population), and change (%) from baseline.
