# Supplementary material for: Estetrol Inhibits the Prostate Cancer Tumor Stimulators FSH and IGF-1
Source: J Clin Med. 2024 Oct 8;13(19):5996. doi: 10.3390/jcm13195996 (PMC11478095; doi:10.3390/jcm13195996)
Supplement: Supplementary file 1 [file jcm-13-05996-s001.zip › jcm-3170539-supplementary.pdf]

Estetrol Inhibits the Prostate Cancer Tumor Stimulators IGF-1 and FSH

Herjan J.T. Coelingh Bennink, et al

Supplementary Data and Information

Table of Contents

	Page
Protocol PR3109 (frontpage)<br>4.4 Patient selection (Inclusion and Exclusion criteria)	2
Figure S1 - Disposition of patients (CONSORT)	4
Figure S2 - Individual change (%) from baseline of total testosterone levels at Weeks 2, 6, 12 and 24 of treatment with 40 mg estetrol or placebo ADT co-administration (per-protocol population)	5
Figure S3 - Individual change (%) from baseline of free testosterone levels at Weeks 2, 6, 12 and 24 of treatment with 40 mg estetrol or placebo ADT co-administration (per-protocol population)	5
Figure S4 - Individual change (%) from baseline of prostate-specific antigen levels at Weeks 2, 6, 12 and 24 of treatment with 40 mg estetrol or placebo ADT co-administration (per-protocol population)	6
Table S1 – Side effects of androgen deprivation therapy with, in brackets, whether it is due to the loss of testosterone (T) or estrogens (E).	7
Table S2 - Individual total testosterone (T) levels throughout the study (per-protocol population), and change (%) from baseline	8
Table S3 - Individual free testosterone (T) levels throughout the study (per-protocol population), and change (%) from baseline	9
Table S4 - Individual prostate-specific antigen (PSA) levels throughout the study (per-protocol population), and change (%) from baseline	10
Table S5 - Individual follicle-stimulating hormone (FSH) levels throughout the study (per-protocol population), and change (%) from baseline	11
Table S6 - Individual insulin-like growth factor-1 (IGF-1) levels throughout the study (per-protocol population), and change (%) from baseline	12

PANTARHEI ONCOLOGY

PROTOCOL PR3109

A double-blind, randomised, placebo-controlled, multi-center study to evaluate effects of estetrol on testosterone suppression and quality of life in prostate cancer patients treated with an LHRH agonist

**Investigational Product:**	**Estetrol**
**Sponsor:**	Pantarhei Oncology BV<br>Boslaan 11<br>3701 CH Zeist<br>Telephone: +31 30 6985020<br>Telefax: +31 30 6985021
**Clinical study protocol:**	PCombi
**Protocol number:**	PR3109
**EudraCT number:**	2017-003708-34
**Version number:**	2.0 Final
**Version date:**	08 November, 2018

4.4.2 Inclusion criteria

In order to be eligible to participate in this study, a patient must meet all of the following criteria:

1. Male patients with prostate cancer, qualifying for treatment with a LHRH agonist;
2. Age ≥ 18 years;
3. Body mass index (BMI) between ≥ 18.0 and ≤ 35.0 kg/m^2 (inclusive);
4. Reasonable physical and mental health as judged by the Investigator determined by physical examination, clinical laboratory assessments and vital signs;
5. Eastern Cooperative Oncology Group (ECOG) Performance Status 0-1;
6. Life expectancy of at least 2 years;
7. Willing to give informed consent in writing.

4.4.3 Exclusion criteria

A potential patient who meets any of the following criteria will be excluded from participation in this study:

1. Current or prior (during the last 12 months) hormonal therapy, immunotherapy or chemotherapy for prostate cancer. Allowed are 14 days concomitant treatment with an anti-androgen to prevent the flare-up, radiotherapy and low dose radiation to prevent gynecomastia;
2. History of deep vein thrombosis, pulmonary embolism, or cerebrovascular accident. However, patients with such history using anticoagulants for ≥ 6 months are eligible for the study provided anticoagulant treatment is continued throughout the whole study;
3. History of myocardial infarction or a coronary vascular procedure (e.g. percutaneous coronary intervention, coronary artery bypass graft). However, patients with such history using anticoagulants for ≥ 6 months are eligible for the study provided anticoagulant treatment is continued throughout the whole study;
4. Patients who have unstable angina or clinical congestive heart failure;
5. A defect in the blood coagulation system, assessed at screening: deficiencies in AT-III, protein C and protein S and elevated factor VIII;
6. Mutation in coagulation factor II and/or positive for factor V Leiden, assessed at screening;
7. Diabetes mellitus with poor glycaemic control in the past 6 months (haemoglobin A1c (HbA1c) above 7.5%);
8. Known primary hyperlipidaemias (Fredrickson);
9. Disturbance of liver function: cholestatic jaundice, a history of jaundice due to previous estrogen use, Rotor syndrome and Dubin-Johnson syndrome;
10. Known porphyria;
11. Uncontrolled hypertension, i.e. systolic blood pressure >160 mmHg and/or diastolic blood pressure >100 mmHg in the last 6 months with or without medication;
12. Subjects with a history of (within 12 months) alcohol or drug abuse;
13. Administration of any other investigational drug within 4 weeks prior to start treatment;
14. Any other condition (e.g. presence of any other malignancy), which in the Investigator's opinion, would not make the patient a good candidate for the trial.

4.4.4 Informed consent

The principles of informed consent will be implemented in accordance with the ethical principles that have their origin in the Declaration of Helsinki, the International Conference on Harmonization (ICH) Guideline for Good Clinical Practice (GCP), and applicable regulatory requirements.

Figure S1 Disposition of patients (CONSORT)

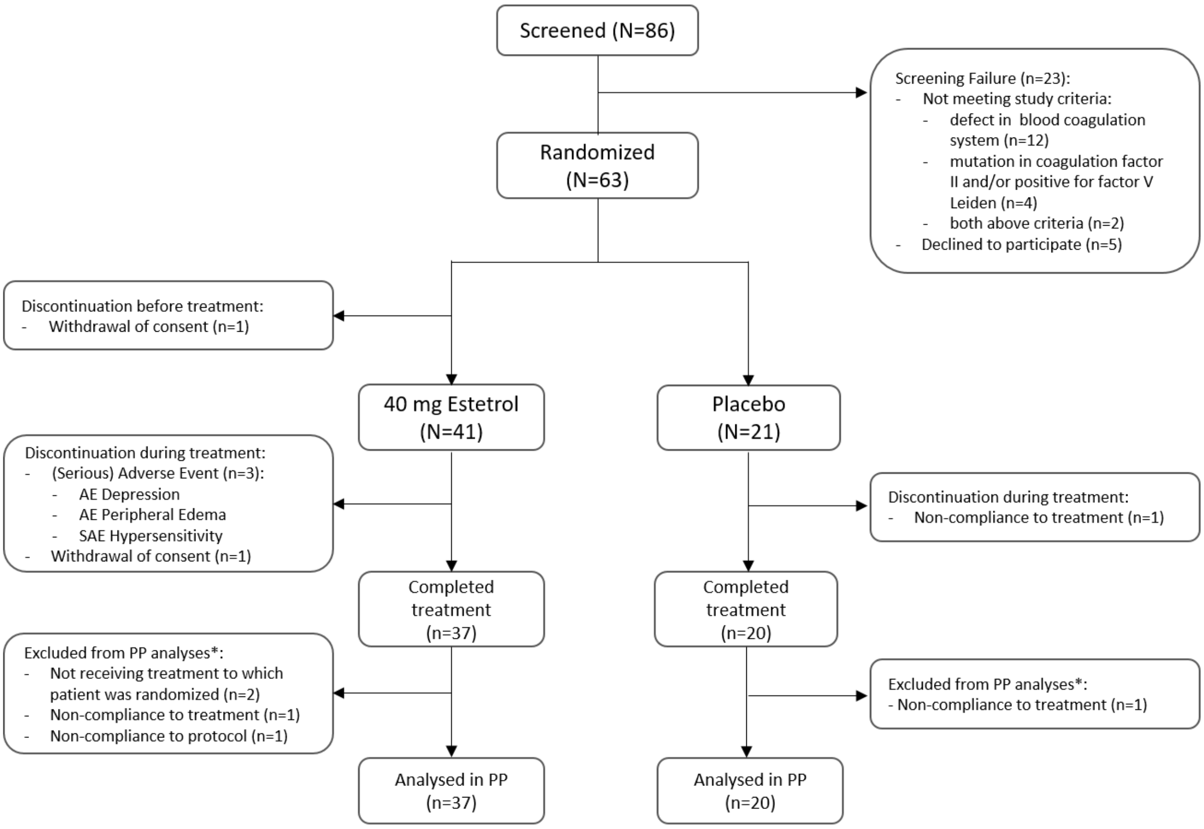

* Based on all patients who started treatment; patients not completing study treatment could still be included in PP analyses if all study assessments were performed (e.g. end of study visit). This was applicable for the patients of the estetrol group, but not for the patient of the placebo group. All patients who received at least one dose of study medication were included in the analyses of the safety parameters

Coelingh Bennink HJT, et al. Eur Urol Open Sci 2021;28:52-61 (CC-BY-NC-ND)

Figure S2 Individual change (%) from baseline of total testosterone levels at Weeks 2, 6, 12 and 24 of treatment with 40 mg estetrol or placebo ADT co-administration (per-protocol population)

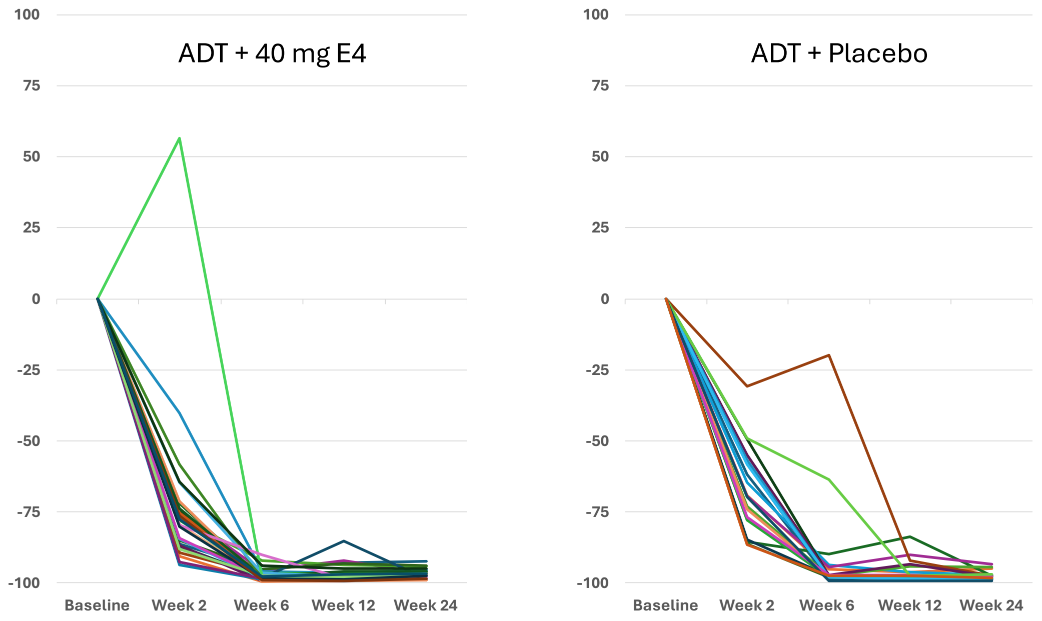

ADT androgen deprivation therapy; E4: estetrol; Source: Supplementary Table S3

Figure S3 Individual change (%) from baseline of free testosterone levels at Weeks 2, 6, 12 and 24 of treatment with 40 mg estetrol or placebo ADT co-administration (per-protocol population)

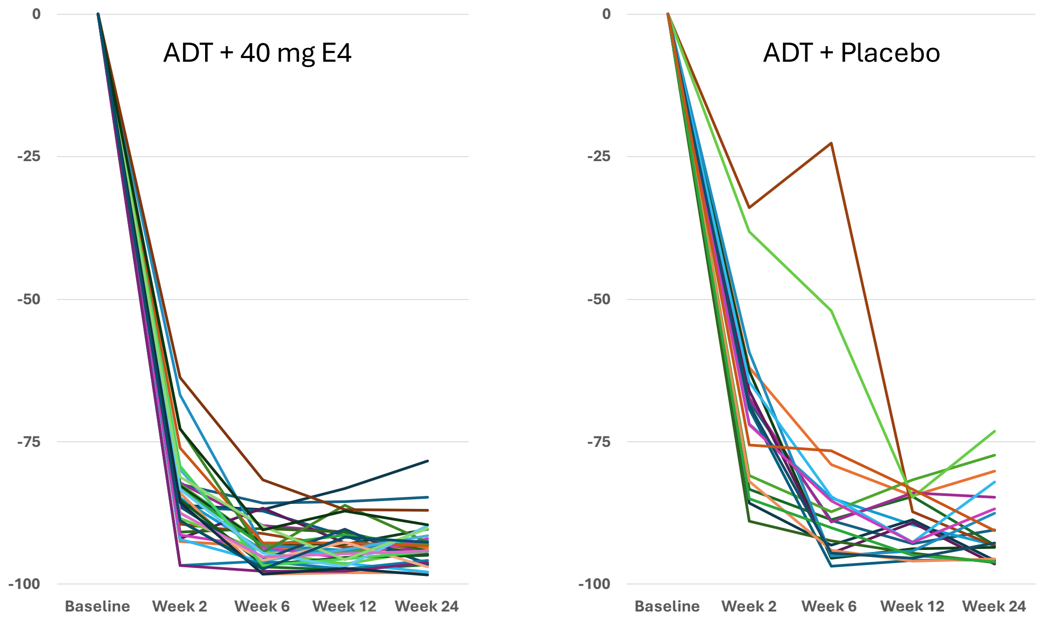

ADT androgen deprivation therapy; E4: estetrol; Source: Supplementary Table S4

Figure S4 Individual change (%) from baseline of prostate-specific antigen levels at Weeks 2, 6, 12 and 24 of treatment with 40 mg estetrol or placebo ADT co-administration (per-protocol population)

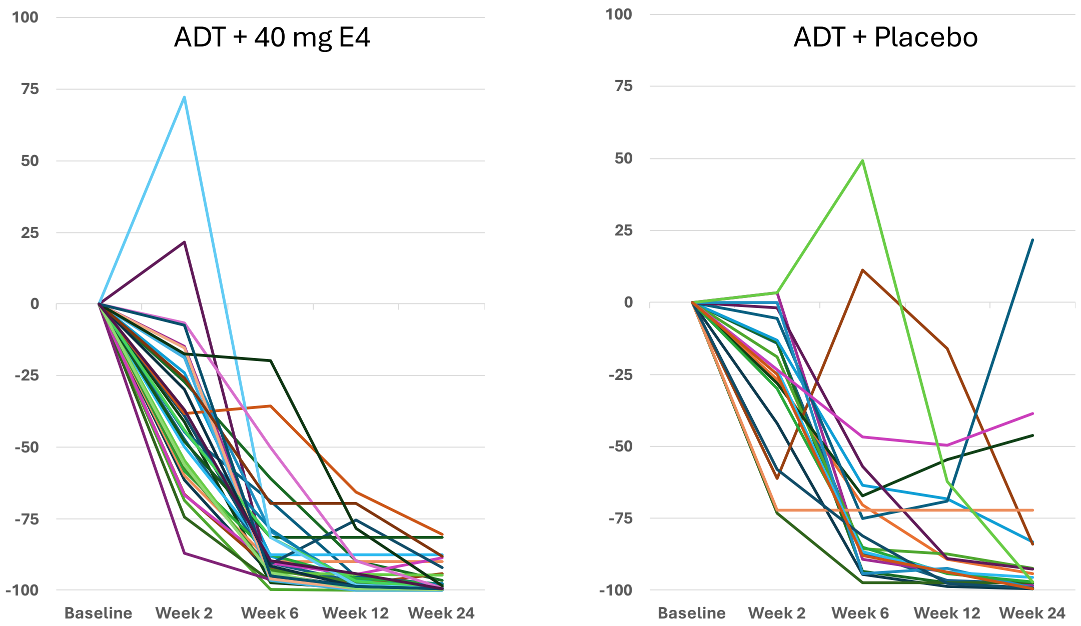

ADT androgen deprivation therapy; E4: estetrol; Source: Supplementary Table S5

Table S1 – Side effects of androgen deprivation therapy with, in brackets, whether it is due to the loss of testosterone (T) or estrogens (E)

**'Big four'**	**What you see**	**What is not visible**	**What the patient feels**
Libido loss **(T)**	Weight gain **(E)**	Loss of bone, decreased bone mineral density and increased fracture risk **(E)**	Fatigue (**T & E)**
Erection problems **(T)**	Gynecomastia **(T & E)**	Metabolic syndrome **(E)**	Sleeping problems **(T & E)**
Hot flushes & sweating **(E)**	Muscle atrophy (sarcopenia) **(T & E)**	Anemia **(T)**	Loss of energy **(T & E)**
Arthralgia (joint pain); **(E)**	Decreased size penis and testicles **(T)**	Increased cardiovascular risk **(loss of E)**	Apathy **(T & E)**
		Inhibition of spermatogenesis and infertility (T)	
	Change hair pattern **(T)**		Mood changes and depression **(E)**
			Cognition and memory problems **(E)**

Adapted from Coelingh Bennink et al. 2022 (reproduced according to BY-NC-ND 4.0)

Table S2 - Individual follicle-stimulating hormone (FSH) levels throughout the study (per-protocol population), and change (%) from baseline

		FSH (IU/L)*			Change vs BL	
**Subject ID**	**Treatment**	**Baseline**	**Week 12**	**Week 24**	**Week 12**	**Week 24**
103-001	40 mg E4	25,6	1,7	0,6	-93,4%	-97,7%
103-003	40 mg E4	9,9	0,1	0,1	-99,0%	-99,0%
103-004	40 mg E4	3,7	0,4	0,2	-89,2%	-94,6%
103-007	40 mg E4	5,2	0,1		-98,1%	
103-008	40 mg E4	1,6	0,05	0,15	-96,9%	-90,6%
103-011	40 mg E4	10,6	0,9	0,3	-91,5%	-97,2%
103-014	40 mg E4	5,0	0,15	0,15	-97,0%	-97,0%
103-016	40 mg E4	60,9	0,15	0,15	-99,8%	-99,8%
103-017	40 mg E4	11,1	0,15	0,15	-98,6%	-98,6%
103-020	40 mg E4	14,8	0,15	0,15	-99,0%	-99,0%
103-028	40 mg E4	4,0	0,15	0,15	-96,3%	-96,3%
103-030	40 mg E4	4,1	0,15	0,15	-96,3%	-96,3%
103-032	40 mg E4	40,2	0,15	0,15	-99,6%	-99,6%
104-002	40 mg E4	5,9	0,2	0,05	-96,6%	-99,2%
104-003	40 mg E4	4,6	0,3	0,05	-93,5%	-98,9%
104-005	40 mg E4	15,1	0,05	0,15	-99,7%	-99,0%
104-006	40 mg E4	8,3				
104-010	40 mg E4	10,7	0,3	0,15	-97,2%	-98,6%
104-011	40 mg E4	13,4	0,2	0,15	-98,5%	-98,9%
104-012	40 mg E4	12,5	0,05	0,15	-99,6%	-98,8%
104-014	40 mg E4	11,1	0,6	0,15	-94,6%	-98,6%
104-017	40 mg E4	4,4	0,15	0,15	-96,6%	-96,6%
104-020	40 mg E4	14,1	0,15	0,15	-98,9%	-98,9%
104-021	40 mg E4	3,7	0,15	0,15	-95,9%	-95,9%
104-023	40 mg E4	5,0	0,15	0,15	-97,0%	-97,0%
104-028	40 mg E4	8,8	0,15	0,15	-98,3%	-98,3%
107-001	40 mg E4	6,4	0,15	0,15	-97,7%	-97,7%
107-004	40 mg E4	8,8	0,15	0,15	-98,3%	-98,3%
107-005	40 mg E4	4,4	0,15	0,15	-96,6%	-96,6%
107-006	40 mg E4	4,6	0,15	0,15	-96,7%	-96,7%
108-004	40 mg E4	5,1	0,15	0,15	-97,1%	-97,1%
108-005	40 mg E4	8,5	0,15	0,15	-98,2%	-98,2%
108-007	40 mg E4	11,4	0,15	0,15	-98,7%	-98,7%
108-010	40 mg E4	10,8	0,15	0,15	-98,6%	-98,6%
108-011	40 mg E4	7,2	0,15	0,15	-97,9%	-97,9%
108-012	40 mg E4	7,4	0,3	0,15	-95,9%	-98,0%
108-014	40 mg E4	27,9	0,15	0,15	-99,5%	-99,5%
103-002	Placebo	12,1	14,5	16,1	19,8%	33,1%
103-006	Placebo	13,9	4,4	4,0	-68,3%	-71,2%
103-010	Placebo	2,9	4,5	4,2	55,2%	44,8%
103-013	Placebo	6,7	2,3	2,5	-65,7%	-62,7%
103-018	Placebo	6,7	6,6	5,6	-1,5%	-16,4%
103-025	Placebo	11,4	2,1	2,3	-81,6%	-79,8%
103-027	Placebo	8,8	4,3	6,5	-51,1%	-26,1%
104-004	Placebo	35,8	3,0	5,6	-91,6%	-84,4%
104-007	Placebo	3,2	4,2	3,9	31,3%	21,9%
104-009	Placebo	2,6	4,3	4,2	65,4%	61,5%
104-016	Placebo	16,2	5,7	5,6	-64,8%	-65,4%
104-019	Placebo	31,0	4,2	5,2	-86,5%	-83,2%
104-024	Placebo	5,8	3,1	3,9	-46,6%	-32,8%
104-027	Placebo	9,3	4,1	4,1	-55,9%	-55,9%
104-030	Placebo	10,1	6,0	6,1	-40,6%	-39,6%
107-002	Placebo	5,1	3,4	3,8	-33,3%	-25,5%
108-002	Placebo	14,6	7,4	5,9	-49,3%	-59,6%
108-008	Placebo	24,6	2,8	3,7	-88,6%	-85,0%
108-009	Placebo	8,6	2,4	3,9	-72,1%	-54,7%
108-013	Placebo	25,4	9,1	11,8	-64,2%	-53,5%

* Imputation rules: FSH <0,1 IU/L = 0,05; <0,3 IU/L = 0,15 (marked in red)

(yellow)	Change vs baseline -75,0% to - 94,9%
(orange)	Change vs baseline ≥- 95.0%

Table S3 - Individual insulin-like growth factor-1 (IGF-1) levels throughout the study (per-protocol population), and change (%) from baseline

		IGF-1 (ng/mL)			Change vs BL	
Subject ID	Treatment	Baseline	Week 12	Week 24	Week 12	Week 24
103-001	40 mg E4		130.8	104.06		
103-003	40 mg E4	177,2				
103-004	40 mg E4	122,5	59,0	44,0	-51,8%	-64,1%
103-007	40 mg E4	193,0	144,8	283,0	-25,0%	46,6%
103-008	40 mg E4	147,6	89,8	59,7	-39,2%	-59,6%
103-011	40 mg E4	200,6	130,1	115,6	-35,1%	-42,4%
103-014	40 mg E4	239,2	111,4	89,9	-53,4%	-62,4%
103-016	40 mg E4	141,0	80,2	88,9	-43,1%	-37,0%
103-017	40 mg E4	154,3	83,9	95,8	-45,6%	-37,9%
103-020	40 mg E4	211,1	105,7	93,1	-49,9%	-55,9%
103-028	40 mg E4	142,4	56,0	85,1	-60,7%	-40,2%
103-030	40 mg E4	168,8	178,0	91,7	5,5%	-45,7%
103-032	40 mg E4	110,8	82,0	102,3	-26,0%	-7,7%
104-002	40 mg E4	192,0	84,4	83,6	-56,0%	-56,5%
104-003	40 mg E4	110,4	71,3		-35,4%	
104-005	40 mg E4	167,8	106,1	108,5	-36,8%	-35,3%
104-006	40 mg E4	164,7	109,7	104,5	-33,4%	-36,6%
104-010	40 mg E4	203,9	115,1	98,2	-43,6%	-51,8%
104-011	40 mg E4	194,9	176,3	134,2	-9,5%	-31,1%
104-012	40 mg E4	225,6	133,7	87,7	-40,7%	-61,1%
104-014	40 mg E4	76,1	91,3	73,4	20,0%	-3,5%
104-017	40 mg E4	204,3	173,9	167,9	-14,9%	-17,8%
104-020	40 mg E4	197,3	109,9	109,4	-44,3%	-44,6%
104-021	40 mg E4	201,8	90,2	105,3	-55,3%	-47,8%
104-023	40 mg E4	166,6	74,3	65,2	-55,4%	-60,9%
104-028	40 mg E4	108,2	99,0	75,9	-8,5%	-29,9%
107-001	40 mg E4	177,9	90,6	96,3	-49,1%	-45,9%
107-004	40 mg E4	212,8	127,0	116,2	-40,3%	-45,4%
107-005	40 mg E4	211,9	174,5	133,4	-17,6%	-37,0%
107-006	40 mg E4	231,8	107,5	117,8	-53,6%	-49,2%
108-004	40 mg E4	133,1	100,7	99,0	-24,3%	-25,6%
108-005	40 mg E4	189,2	89,7	120,9	-52,6%	-36,1%
108-007	40 mg E4	172,4	123,7	117,9	-28,2%	-31,6%
108-010	40 mg E4	187,8	130,4	104,8	-30,6%	-44,2%
108-011	40 mg E4	183,2	99,5	86,3	-45,7%	-52,9%
108-012	40 mg E4	198,5	168,3	160,4	-15,2%	-19,2%
108-014	40 mg E4	144,7	66,2	79,7	-54,3%	-44,9%
103-002	Placebo	213,0	265,6	249,9	24,7%	17,3%
103-006	Placebo	184,0	175,0	264,1	-4,9%	43,5%
103-010	Placebo	116,0	101,4	109,3	-12,6%	-5,8%
103-013	Placebo	118,1	90,1	105,9	-23,7%	-10,3%
103-018	Placebo	112,4	126,7	124,9	12,7%	11,1%
103-025	Placebo	130,0	120,4	118,1	-7,4%	-9,2%
103-027	Placebo	110,0	91,5	102,8	-16,8%	-6,5%
104-004	Placebo	168,6	143,4	86,9	-14,9%	-48,5%
104-007	Placebo	146,6	94,8	124,3	-35,3%	-15,2%
104-009	Placebo	191,9	279,9	313,9	45,9%	63,6%
104-016	Placebo	147,5	93,2	126,5	-36,8%	-14,2%
104-019	Placebo	137,5	223,6	175,6	62,6%	27,7%
104-024	Placebo	153,1	132,1	179,0	-13,7%	16,9%
104-027	Placebo	285,0	486,7	398,8	70,8%	39,9%
104-030	Placebo	164,4	137,1	135,0	-16,6%	-17,9%
107-002	Placebo	85,9	108,5	148,5	26,3%	72,9%
108-002	Placebo	119,4	141,8	156,5	18,8%	31,1%
108-008	Placebo	104,0	95,6	85,6	-8,1%	-17,7%
108-009	Placebo	175,8	161,7	219,5	-8,0%	24,9%
108-013	Placebo	198,4	157,2	198,8	-20,8%	0,2%

Colour	Legend
Yellow	Change vs baseline -10,0% to -24,9%
Orange	Change vs baseline -25,0% to -49,9%
Red	Change vs baseline ≥-50,0%

Table S4 - Individual total testosterone (T) levels throughout the study (per-protocol population), and change (%) from baseline

		Total Testosterone (pmol/L)*,**					Change vs BL			
**Subject ID**	**Treatment**	**Baseline**	**Week 2**	**Week 6**	**Week 12**	**Week 24**	**Week 2**	**Week 6**	**Week 12**	**Week 24**
103-001	40 mg E4	9,98	1,38	0,47	0,71	0,77	-86,2%	-95,3%	-92,9%	-92,3%
103-003	40 mg E4	16,90	1,57	0,09	0,22	0,17	-90,7%	-99,5%	-98,7%	-99,0%
103-004	40 mg E4	25,30	7,07	0,43	0,44	0,59	-72,1%	-98,3%	-98,3%	-97,7%
103-007	40 mg E4	11,90	2,69	0,47	0,50		-77,4%	-96,1%	-95,8%	
103-008	40 mg E4	18,90	4,54	0,36	0,40	0,31	-76,0%	-98,1%	-97,9%	-98,4%
103-011	40 mg E4	15,00	3,78	0,66	1,18	0,50	-74,8%	-95,6%	-92,1%	-96,7%
103-014	40 mg E4	10,30	2,36	0,82	0,66	0,61	-77,1%	-92,0%	-93,6%	-94,1%
103-016	40 mg E4	9,43	1,23	0,18	0,24	0,22	-87,0%	-98,1%	-97,5%	-97,7%
103-017	40 mg E4	18,00	1,92	0,53	0,52	0,65	-89,3%	-97,1%	-97,1%	-96,4%
103-020	40 mg E4	12,90	3,30	0,27	0,33	0,58	-74,4%	-97,9%	-97,4%	-95,5%
103-028	40 mg E4	20,30	4,61	0,52	0,53	0,33	-77,3%	-97,4%	-97,4%	-98,4%
103-030	40 mg E4	14,20	1,89	0,09	0,09	0,19	-86,7%	-99,4%	-99,4%	-98,7%
103-032	40 mg E4	15,70	2,35	0,74	1,09	0,94	-85,0%	-95,3%	-93,1%	-94,0%
104-002	40 mg E4	13,50	8,08	0,56	0,47	0,61	-40,1%	-95,9%	-96,5%	-95,5%
104-003	40 mg E4	32,50	9,36	0,18	0,28	0,29	-71,2%	-99,4%	-99,1%	-99,1%
104-005	40 mg E4	10,90	2,42	0,40	0,37	0,43	-77,8%	-96,3%	-96,6%	-96,1%
104-006	40 mg E4	54,10	12,90	0,78			-76,2%	-98,6%		
104-010	40 mg E4	26,90	4,20	0,48	0,50	0,58	-84,4%	-98,2%	-98,1%	-97,8%
104-011	40 mg E4	16,50	2,61	0,35	0,44	0,46	-84,2%	-97,9%	-97,3%	-97,2%
104-012	40 mg E4	24,50	4,99	0,55	0,67	0,81	-79,6%	-97,8%	-97,3%	-96,7%
104-014	40 mg E4	22,60	5,44	0,40	3,35	0,34	-75,9%	-98,2%	-85,2%	-98,5%
104-017	40 mg E4	17,50	4,30	0,48	0,49	0,52	-75,4%	-97,3%	-97,2%	-97,0%
104-020	40 mg E4	26,70	6,97	0,62	1,08	1,06	-73,9%	-97,7%	-96,0%	-96,0%
104-021	40 mg E4	31,00	1,99	0,37	0,52	0,78	-93,6%	-98,8%	-98,3%	-97,5%
104-023	40 mg E4	25,60	1,86	0,39	0,41	0,37	-92,7%	-98,5%	-98,4%	-98,6%
104-028	40 mg E4	23,00	9,60	0,38	0,44	0,49	-58,3%	-98,3%	-98,1%	-97,9%
107-001	40 mg E4	21,10	7,49	0,73	0,47	0,68	-64,5%	-96,5%	-97,8%	-96,8%
107-004	40 mg E4	19,40	3,99	0,31	0,52	0,36	-79,4%	-98,4%	-97,3%	-98,1%
107-005	40 mg E4	11,30	17,70	0,26	0,35	0,38	56,6%	-97,7%	-96,9%	-96,6%
107-006	40 mg E4	11,00	2,50	0,23	0,09	0,19	-77,3%	-97,9%	-99,2%	-98,3%
108-004	40 mg E4	16,60	3,36	1,63	0,25	0,32	-79,8%	-90,2%	-98,5%	-98,1%
108-005	40 mg E4	15,60	1,86	0,39	0,30	0,52	-88,1%	-97,5%	-98,1%	-96,7%
108-007	40 mg E4	17,60	3,50	0,27	0,24	0,44	-80,1%	-98,5%	-98,6%	-97,5%
108-010	40 mg E4	30,50	7,21	0,31	0,25	0,45	-76,4%	-99,0%	-99,2%	-98,5%
108-011	40 mg E4	13,60	4,85	0,84	0,68	0,68	-64,3%	-93,8%	-95,0%	-95,0%
108-012	40 mg E4	17,60	3,92	0,39	0,52	0,57	-77,7%	-97,8%	-97,0%	-96,8%
108-014	40 mg E4	20,70	2,70	0,20	0,19	0,18	-87,0%	-99,0%	-99,1%	-99,1%
103-002	Placebo	15,40	5,86	0,23	0,18	0,20	-61,9%	-98,5%	-98,8%	-98,7%
103-006	Placebo	6,73	2,08	0,33	0,25	0,34	-69,1%	-95,1%	-96,3%	-94,9%
103-010	Placebo	15,60	2,28	1,57	2,55	0,38	-85,4%	-89,9%	-83,7%	-97,6%
103-013	Placebo	20,40	7,21	1,28	0,77	0,59	-64,7%	-93,7%	-96,2%	-97,1%
103-018	Placebo	15,40	4,72	0,86	1,53	1,03	-69,4%	-94,4%	-90,1%	-93,3%
103-025	Placebo	14,60	3,92	0,36	0,87	0,83	-73,2%	-97,5%	-94,0%	-94,3%
103-027	Placebo	22,10	3,39	0,55	0,24	0,28	-84,7%	-97,5%	-98,9%	-98,7%
104-004	Placebo	19,20	13,30	15,40	1,50	0,50	-30,7%	-19,8%	-92,2%	-97,4%
104-007	Placebo	17,30	8,77	0,46	0,39	0,48	-49,3%	-97,3%	-97,7%	-97,2%
104-009	Placebo	31,20	13,10	0,44	0,65	0,48	-58,0%	-98,6%	-97,9%	-98,5%
104-016	Placebo	23,30	10,50	0,67	1,53	0,55	-54,9%	-97,1%	-93,4%	-97,6%
104-019	Placebo	20,40	2,79	0,42	0,49	0,48	-86,3%	-97,9%	-97,6%	-97,6%
104-024	Placebo	20,00	8,67	0,42	0,42	0,30	-56,7%	-97,9%	-97,9%	-98,5%
104-027	Placebo	23,90	6,21	0,48	0,38	0,48	-74,0%	-98,0%	-98,4%	-98,0%
104-030	Placebo	28,60	6,31	0,56	0,56	0,48	-77,9%	-98,0%	-98,0%	-98,3%
107-002	Placebo	12,30	5,13	0,26	0,20	0,21	-58,3%	-97,9%	-98,4%	-98,3%
108-002	Placebo	12,40	2,86	0,35	0,35	0,28	-76,9%	-97,2%	-97,2%	-97,7%
108-008	Placebo	16,20	8,25	5,89	0,43	0,50	-49,1%	-63,6%	-97,3%	-96,9%
108-009	Placebo	32,70	9,88	0,28	0,24	0,28	-69,8%	-99,1%	-99,3%	-99,1%
108-013	Placebo	14,10	1,89	0,36	0,35	0,26	-86,6%	-97,4%	-97,5%	-98,2%

* Imputation rules: Total T <0,173 nmol/L = 0,09 (marked in red)

** Profound castration level <0,693 nmol/L

Change vs baseline -75,0% to - 94,9%

Change vs baseline ≥- 95.0%

Table S5 - Individual free testosterone (T) levels throughout the study (per-protocol population), and change (%) from baseline

		Free Testosterone (pmol/L)					Change vs BL			
Subject ID	Treatment	Baseline	Week 2	Week 6	Week 12	Week 24	Week 2	Week 6	Week 12	Week 24
103-001	40 mg E4	18,17	3,19	2,60	2,64	2,77	-82,4%	-85,7%	-85,5%	-84,8%
103-003	40 mg E4	23,82	1,80	1,59	1,39	1,53	-92,4%	-93,3%	-94,2%	-93,6%
103-004	40 mg E4	43,41	4,82	1,35	1,11	1,49	-88,9%	-96,9%	-97,4%	-96,6%
103-007	40 mg E4		5,27	2,81	2,18					
103-008	40 mg E4	44,14	6,69	2,64	2,50	2,36	-84,8%	-94,0%	-94,3%	-94,7%
103-011	40 mg E4	32,73	5,82	3,36	2,98	1,70	-82,2%	-89,7%	-90,9%	-94,8%
103-014	40 mg E4	23,51	4,13	1,70	1,32	1,66	-82,4%	-92,8%	-94,4%	-92,9%
103-016	40 mg E4	19,55	2,70	2,57	3,29	4,23	-86,2%	-86,9%	-83,2%	-78,4%
103-017	40 mg E4	30,48	3,22	2,70	1,98	2,08	-89,4%	-91,1%	-93,5%	-93,2%
103-020	40 mg E4	25,07	4,33	1,80	1,77	2,43	-82,7%	-92,8%	-92,9%	-90,3%
103-028	40 mg E4	31,20	4,23	4,02	2,67	1,14	-86,4%	-87,1%	-91,4%	-96,3%
103-030	40 mg E4	25,55	2,05	3,40	1,84	1,32	-92,0%	-86,7%	-92,8%	-94,8%
103-032	40 mg E4	33,94	3,09	3,33	3,12	1,98	-90,9%	-90,2%	-90,8%	-94,2%
104-002	40 mg E4	33,49	11,13	2,32	2,01	2,57	-66,8%	-93,1%	-94,0%	-92,3%
104-003	40 mg E4	71,25	11,41	1,28	1,49	1,46	-84,0%	-98,2%	-97,9%	-98,0%
104-005	40 mg E4	34,46	5,79	2,32	2,98	2,39	-83,2%	-93,3%	-91,4%	-93,1%
104-006	40 mg E4	76,69	9,85	3,29			-87,2%	-95,7%		
104-010	40 mg E4	70,11	5,51	2,77	2,57	1,49	-92,1%	-96,0%	-96,3%	-97,9%
104-011	40 mg E4	33,53	2,91	2,18	1,53	2,70	-91,3%	-93,5%	-95,4%	-91,9%
104-012	40 mg E4	71,70	8,32	4,06	2,53	4,26	-88,4%	-94,3%	-96,5%	-94,1%
104-014	40 mg E4	51,00	6,93	2,22	4,96	1,73	-86,4%	-95,6%	-90,3%	-96,6%
104-017	40 mg E4	38,07	9,12	2,74	2,77	2,57	-76,0%	-92,8%	-92,7%	-93,2%
104-020	40 mg E4	62,72	9,36	2,15	2,95	3,19	-85,1%	-96,6%	-95,3%	-94,9%
104-021	40 mg E4	70,00	2,29	2,84	1,91	2,95	-96,7%	-95,9%	-97,3%	-95,8%
104-023	40 mg E4	62,86	2,11	1,42	1,46	2,39	-96,6%	-97,7%	-97,7%	-96,2%
104-028	40 mg E4	41,95	11,48	2,36	5,82	3,12	-72,6%	-94,4%	-86,1%	-92,6%
107-001	40 mg E4	38,83	7,80	1,63	2,36	3,29	-79,9%	-95,8%	-93,9%	-91,5%
107-004	40 mg E4	49,72	6,28	2,08	3,68	1,53	-87,4%	-95,8%	-92,6%	-96,9%
107-005	40 mg E4	32,21	36,93	2,29		1,59	14,7%	-92,9%		-95,1%
107-006	40 mg E4	24,86	5,17	0,83	1,11	1,42	-79,2%	-96,7%	-95,5%	-94,3%
108-004	40 mg E4	40,25	6,69	2,18	1,25	4,16	-83,4%	-94,6%	-96,9%	-89,7%
108-005	40 mg E4	43,10	5,37	2,11	2,29	2,53	-87,5%	-95,1%	-94,7%	-94,1%
108-007	40 mg E4	30,30	5,69	3,09	1,32	2,95	-81,2%	-89,8%	-95,6%	-90,3%
108-010	40 mg E4	79,47	11,44	1,42	2,18	1,35	-85,6%	-98,2%	-97,3%	-98,3%
108-011	40 mg E4	38,62	14,01	7,07	5,06	5,03	-63,7%	-81,7%	-86,9%	-87,0%
108-012	40 mg E4	31,86	8,67	3,02	4,09	3,33	-72,8%	-90,5%	-87,2%	-89,5%
108-014	40 mg E4	34,91	3,92	0,94	2,88	2,57	-88,8%	-97,3%	-91,8%	-92,6%
103-002	Placebo	22,95	7,35	2,57	1,63	2,18	-68,0%	-88,8%	-92,9%	-90,5%
103-006	Placebo	16,57	6,31	3,47	2,57	3,29	-61,9%	-79,1%	-84,5%	-80,1%
103-010	Placebo	30,20	5,03	3,43	4,65	2,08	-83,3%	-88,6%	-84,6%	-93,1%
103-013	Placebo	33,42	9,40	5,06	3,47	2,29	-71,9%	-84,9%	-89,6%	-93,1%
103-018	Placebo	23,16	7,56	2,53	3,71	3,54	-67,4%	-89,1%	-84,0%	-84,7%
103-025	Placebo	31,52	6,03	4,02	5,79	7,14	-80,9%	-87,2%	-81,6%	-77,3%
103-027	Placebo	41,71	5,96	2,88	4,72	1,77	-85,7%	-93,1%	-88,7%	-95,8%
104-004	Placebo	44,69	29,54	34,57	5,69	3,02	-33,9%	-22,6%	-87,3%	-93,2%
104-007	Placebo	48,85	18,27	2,22	3,05	3,19	-62,6%	-95,5%	-93,8%	-93,5%
104-009	Placebo	71,84	22,09	2,32	3,05	3,12	-69,3%	-96,8%	-95,8%	-95,7%
104-016	Placebo	29,57	10,05	1,63	3,19	1,07	-66,0%	-94,5%	-89,2%	-96,4%
104-019	Placebo	49,27	5,48	3,78	2,67	1,87	-88,9%	-92,3%	-94,6%	-96,2%
104-024	Placebo	54,43	22,16	2,70	3,19	6,76	-59,3%	-95,0%	-94,1%	-87,6%
104-027	Placebo	64,84	11,68	3,85	2,67	2,88	-82,0%	-94,1%	-95,9%	-95,6%
104-030	Placebo	52,11	7,80	5,20	2,64	2,08	-85,0%	-90,0%	-94,9%	-96,0%
107-002	Placebo	20,28	7,21	3,12	1,49	3,64	-64,4%	-84,6%	-92,7%	-82,1%
108-002	Placebo	27,88	7,80	4,09	2,05	3,68	-72,0%	-85,3%	-92,6%	-86,8%
108-008	Placebo	20,01	12,38	9,60	3,05	5,37	-38,1%	-52,0%	-84,8%	-73,2%
108-009	Placebo	43,96	13,80	2,43	2,01	3,16	-68,6%	-94,5%	-95,4%	-92,8%
108-013	Placebo	26,70	6,52	6,24	4,44	2,53	-75,6%	-76,6%	-83,4%	-90,5%

(yellow)	Change vs baseline -75,0% to - 94,9%
(orange)	Change vs baseline ≥- 95.0%

Table S6 - Individual prostate-specific antigen (PSA) levels throughout the study (per-protocol population), and change (%) from baseline

		PSA (ng/mL)*					Change vs BL			
**Subject ID**	**Treatment**	**Baseline**	**Week 2**	**Week 6**	**Week 12**	**Week 24**	**Week 2**	**Week 6**	**Week 12**	**Week 24**
103-001	40 mg E4	10,60	5,60	1,00	0,30	0,10	-47,2%	-90,6%	-97,2%	-99,1%
103-003	40 mg E4	34,00	105,3 (a)	3,80	0,30	0,05		-88,8%	-99,1%	-99,9%
103-004	40 mg E4	5,90	4,30	2,30	0,60	0,20	-27,1%	-61,0%	-89,8%	-96,6%
103-007	40 mg E4	24,80	18,50	3,00	1,13		-25,4%	-87,9%	-95,4%	
103-008	40 mg E4	5,00	3,80	0,60	0,20	0,05	-24,0%	-88,0%	-96,0%	-99,0%
103-011	40 mg E4	15,50	13,20	1,15	0,38	0,05	-14,8%	-92,6%	-97,5%	-99,7%
103-014	40 mg E4	44,75	14,03	0,16	0,05	0,05	-68,6%	-99,6%	-99,9%	-99,9%
103-016	40 mg E4	117,80	45,48	6,55	1,57	0,55	-61,4%	-94,4%	-98,7%	-99,5%
103-017	40 mg E4	2,45	0,82	0,18	0,05	0,14	-66,5%	-92,7%	-98,0%	-94,3%
103-020	40 mg E4	17,72	10,41	0,47	0,15	0,05	-41,3%	-97,3%	-99,2%	-99,7%
103-028	40 mg E4	9,29	5,15	2,88	0,44	0,20	-44,6%	-69,0%	-95,3%	-97,8%
103-030	40 mg E4	13,58	16,52	0,57	0,05	0,05	21,6%	-95,8%	-99,6%	-99,6%
103-032	40 mg E4	11,07	2,82	0,34	0,11	0,05	-74,5%	-96,9%	-99,0%	-99,5%
104-002	40 mg E4	12,10	9,90	2,50	0,30	0,05	-18,2%	-79,3%	-97,5%	-99,6%
104-003	40 mg E4	0,50	0,20	0,05	0,05	0,05	-60,0%	-90,0%	-90,0%	-90,0%
104-005	40 mg E4	2,40	1,00	0,30	0,10	0,05	-58,3%	-87,5%	-95,8%	-97,9%
104-006	40 mg E4	0,29	0,05	0,05			-82,8%	-82,8%		
104-010	40 mg E4	0,40	0,20	0,05	0,05	0,05	-50,0%	-87,5%	-87,5%	-87,5%
104-011	40 mg E4	15,90	5,30	1,50	0,88	1,81	-66,7%	-90,6%	-94,5%	-88,6%
104-012	40 mg E4	9,90	4,50	0,60	0,54	0,53	-54,5%	-93,9%	-94,5%	-94,6%
104-014	40 mg E4	7,24	4,41	0,67	1,77	0,58	-39,1%	-90,7%	-75,6%	-92,0%
104-017	40 mg E4	64,80	39,96	41,69	22,29	12,57	-38,3%	-35,7%	-65,6%	-80,6%
104-020	40 mg E4	0,27	0,14	0,05	0,05	0,05	-48,1%	-81,5%	-81,5%	-81,5%
104-021	40 mg E4	27,19	15,09	5,84	0,29	0,66	-44,5%	-78,5%	-98,9%	-97,6%
104-023	40 mg E4	12,27	1,58	0,45	0,14	0,05	-87,1%	-96,3%	-98,9%	-99,6%
104-028	40 mg E4	26,42	11,34	1,86	0,80	0,33	-57,1%	-93,0%	-97,0%	-98,8%
107-001	40 mg E4	39,66	32,26	1,36	0,05	0,05	-18,7%	-96,6%	-99,9%	-99,9%
107-004	40 mg E4	26,40	22,34	1,01	0,14	0,05	-15,4%	-96,2%	-99,5%	-99,8%
107-005	40 mg E4	7,19	4,02	1,33	0,22	0,05	-44,1%	-81,5%	-96,9%	-99,3%
107-006	40 mg E4	12,61	21,73	2,32	0,24	0,05	72,3%	-81,6%	-98,1%	-99,6%
108-004	40 mg E4	6,10	5,70	3,04	0,62	0,05	-6,6%	-50,2%	-89,8%	-99,2%
108-005	40 mg E4	30,49	13,38	1,84	0,13	0,05	-56,1%	-94,0%	-99,6%	-99,8%
108-007	40 mg E4	8,43	5,92	0,72	0,12	0,05	-29,8%	-91,5%	-98,6%	-99,4%
108-010	40 mg E4	2,01	1,49	0,61	0,61	0,24	-25,9%	-69,7%	-69,7%	-88,1%
108-011	40 mg E4	11,35	9,38	9,09	2,46	0,19	-17,4%	-19,9%	-78,3%	-98,3%
108-012	40 mg E4	35,39	32,81	1,80	0,47	0,15	-7,3%	-94,9%	-98,7%	-99,6%
108-014	40 mg E4	8,49	5,35	0,87	0,50	0,05	-37,0%	-89,8%	-94,1%	-99,4%
103-002	Placebo	41,00	35,20	5,40	1,40	1,00	-14,1%	-86,8%	-96,6%	-97,6%
103-006	Placebo	34,71	25,40	10,25	3,70	1,96	-26,8%	-70,5%	-89,3%	-94,4%
103-010	Placebo	12,20	10,50	0,79	0,31	0,05	-13,9%	-93,5%	-97,5%	-99,6%
103-013	Placebo	241,80	209,70	88,46	76,89	40,60	-13,3%	-63,4%	-68,2%	-83,2%
103-018	Placebo	18,62	19,23	2,00	1,17	0,30	3,3%	-89,3%	-93,7%	-98,4%
103-025	Placebo	15,78	12,81	2,28	1,97	1,18	-18,8%	-85,6%	-87,5%	-92,5%
103-027	Placebo	113,40	65,75	6,38	1,54	0,58	-42,0%	-94,4%	-98,6%	-99,5%
104-004	Placebo	6,20	2,40	6,90	5,20	1,00	-61,3%	11,3%	-16,1%	-83,9%
104-007	Placebo	6,40	4,60	2,10	2,90	3,44	-28,1%	-67,2%	-54,7%	-46,3%
104-009	Placebo	9,20	8,70	2,30	2,84	11,20	-5,4%	-75,0%	-69,1%	21,7%
104-016	Placebo	22,61	22,16	9,75	2,50	1,68	-2,0%	-56,9%	-88,9%	-92,6%
104-019	Placebo	1,99	0,53	0,05	0,05	0,05	-73,4%	-97,5%	-97,5%	-97,5%
104-024	Placebo	7,63		0,44	0,58	0,05		-94,2%	-92,4%	-99,3%
104-027	Placebo	0,18	0,05	0,05	0,05	0,05	-72,2%	-72,2%	-72,2%	-72,2%
104-030	Placebo	1,74	1,22	0,26	0,10	0,05	-29,9%	-85,1%	-94,3%	-97,1%
107-002	Placebo	12,03	9,22	1,61	0,76	0,54	-23,4%	-86,6%	-93,7%	-95,5%
108-002	Placebo	26,10	20,00	13,90	13,16	16,03	-23,4%	-46,7%	-49,6%	-38,6%
108-008	Placebo	4,61	4,76	6,88	1,74	0,13	3,3%	49,2%	-62,3%	-97,2%
108-009	Placebo	6,58	2,77	1,25	0,16	0,05	-57,9%	-81,0%	-97,6%	-99,2%
108-013	Placebo	86,56	64,99	10,46	5,38	0,41	-24,9%	-87,9%	-93,8%	-99,5%

* Imputation rules: PSA <0,1 ng/mL = 0,05 (marked in red)

(a) removed from individual graph for clarity reasons, but included in statistical analysis

Change vs baseline -75,0% to - 94,9%

Change vs baseline ≥- 95.0%
